# Complete chloroplast genome sequences of two endangered *Phoebe* (Lauraceae) species

**DOI:** 10.1186/s40529-017-0192-8

**Published:** 2017-09-13

**Authors:** Yingang Li, Wuqin Xu, Wentao Zou, Dongyue Jiang, Xinhong Liu

**Affiliations:** 1 Zhejiang Academy of Forestry, Hangzhou, 310023 China; 2 Key Laboratory of Conservation Biology for Endangered Wildlife of Ministry of Education, College of Life Sciences, Zhejiang University, Hangzhou, 310058 China; 3 Institute of Tropical Forestry, Chinese Academy of Forestry, Guangzhou, 510520 China

**Keywords:** *Phoebe chekiangensis*, *Phoebe bournei*, Chloroplast genomes, Repeat analysis, SSRs, Divergent regions

## Abstract

**Background:**

*Phoebe* (Lauraceae) comprises of evergreen trees or shrubs with approximately 100 species, distributed in tropical and subtropical Asia and Neotropical America. A total of 34 species and three varieties occur in China. Despite of economic and ecological value, only limited genomic resources are available for this genus.

**Results:**

We sequenced the two complete chloroplast (cp) genomes of *Phoebe chekiangensis* and *P. bournei* using Illumina sequencing technology via a combined strategy of de novo and reference-guided assembly. We also performed comparative analyses with the cp genomes of *P. sheareri* and *P. sheareri* var*. oineiensis* previously reported. The chloroplast genomes of *P. chekiangensis* and *P. bournei* identically contain 112 genes consisting of 78 protein coding genes, 30 tRNA genes, and 4 rRNA genes, with the size of 152,849 and 152,853 bp, respectively. From the two chloroplast genomes, 131 SSRs were identified and 12 different SSRs located in five protein coding genes. The analysis showed the extremely conserved structure of chloroplast genomes with surprisingly little variations at the LSC/IR and SSC/IR boundaries. Moreover, the mean nucleotide diversity was found to be 0.162% for 77 regions, suggesting an extraordinarily low level of sequence divergence. Four highest divergent regions (*trnH*-*psbA, rps14*-*trnT, petA*-*psbJ, ccsA*-*ndhD*) with the percentage of nucleotide diversity higher than 0.50% were identified, which had potential use for species identification and phylogenetic studies.

**Conclusion:**

This study will facilitate our understanding of population genetics, phylogenetic relationship and plant evolution of *Phoebe* species.

**Electronic supplementary material:**

The online version of this article (doi:10.1186/s40529-017-0192-8) contains supplementary material, which is available to authorized users.

## Background

*Phoebe* is a genus of evergreen trees or shrubs belonging to family, Lauraceae. *Phoebe* comprises of approximately 100 species, distributed in tropical and subtropical Asia and neotropical America. A total of 34 species and three varieties are endemic to China (Wu et al. 2008). *Phoebe* species, with their high-quality wood, were widely used to make column during palace construction in the Ming and Qing dynasties and built high valuable furniture which stood for the power and status of the noble (Ding et al. 2015). The most famous and valuable *Phoebe* wood are called ‘wood with golden wire’, which comes from several specific and sporadically distributed rare *Phoebe* species, endemics to China, including *Phoebe chekiangensis*, *Phoebe bournei*, *Phoebe sheareri*, *Phoebe zhennan*, and *Phoebe lichuanensis.* Our target species, *P. chekiangensis* is distributed in Zhejiang and its adjacent areas (including Fujian, Jiangxi, and Anhui Province), whereas *P. bournei* is distributed in Yangtze River Basin and its south region in China. The *Phoebe* species have a very high economic and ecological value but with extremely limited studies on biochemical compound and population diversity (Hegde et al. 1997; Zhang et al. 2012a, b).

The chloroplast (cp) is an important organelle that plays a key role in plant photosynthesis providing energy to green plants and carbon fixation (Douglas 1990). With the rapid development of next-generation sequencing, it is now cheaper and faster to obtain genomes than by traditional Sanger sequencing. Therefore, cp genome-scale data have been increasingly used to infer phylogenetic relationships at high taxonomical levels, and even in lower taxa (Moore et al. 2007; Parks et al. 2009; Huang et al. 2014; Yang et al. 2017; Zeng et al. 2017). Although the cp genome is more conserved than the nuclear genome in plants, many mutation events in the chloroplast DNA sequence have been identified, including indels, substitutions, and inversions (Ingvarsson et al. 2003). Most angiosperm cp genomes have a quadripartite circular structure ranging from 115 to 165 kp in length, and are composed of two copies of inverted repeat (IR) regions that are separated by a large single copy (LSC) region and a small single copy (SSC) region (Wicke et al. 2011; Wang et al. 2015). Massive information for genetics, taxonomy, and phylogeny could be mined from cp genomes, because of their relatively conserved gene structure and sequence divergence between species and individuals (Parks et al. 2009; Huang et al. 2014). Furthermore, cp genomes can provide effective genetic markers for evolutionary studies from population level. In addition, analyzing the composition and structure of the cp genomes for such an important genus *Phoebe* can explore further genetic variations, which could improve quantitative and quality traits.

In this study, we reported the complete and annotated DNA sequences for the cp genomes of *P. chekiangensis* and *P. bournei* using next-generation sequencing platform, which is also the first comprehensive analysis on cp genomes for *Phoebe* combining the cp genomes of *P. sheareri* and *P. sheareri* var*. oineiensis* previously reported (Song et al. 2016). The specific aims of the present study were to: (1) present the complete chloroplast genome sequences and investigate global structural patterns of *P. chekiangensis* and *P. bournei*; (2) examine variations of repeat sequences and simple sequence repeats (SSRs) among the two *Phoebe* chloroplast genomes; (3) screen sequence divergence hotspot regions in the four *Phoebe* chloroplast genomes.

## Methods

### Plant material and DNA extraction

Young leaves of *P. chekiangensis* and *P. bournei* were sampled from single seedlings growing in the nursery located at 30.21N, 120.02E, of Zhejiang Academy of Forestry. The provenance was Hangzhou, Zhejiang Province and Mingxi, Fujian Province, PR China, which was the primary distribution area of *P. chekiangensis* and *P. bournei*, respectively. Total genomic DNA per species was extracted from 30 mg of the silica-dried leaf using the modified CTAB method (Porebski et al. 1997). The quality and concentration of the genomic DNA were assessed using agarose gel electrophoresis and an Agilent BioAnalyzer 2100 (Agilent Technologies).

### Chloroplast genome illumina sequencing, assembly and annotation

Genomic DNA was used to generate short-insert (500 bp) paired-end sequencing libraries according to the Illumina standard protocol. Genomic DNA from each species was sequenced using a HiSeq™ 2000 analyzer (Illumina, San Diego, California, USA) at Beijing Genomics Institute (BGI, Shenzhen, China).The raw reads (20,777,674 and 20,787,108 bp for *P. chekiangensis* and *P. bournei*) were generated with 125 bp length and assembled into whole chloroplast genomes in a multi-step approach employing a modified pipeline that involved a combination of both reference guided and de novo assembly approaches. First, paired-end sequence reads were trimmed to remove low-quality bases (*Q* <20, 0.01 probability error) and adapter sequences using CLC-quality trim tool (quality_trim software included in CLC ASSEMBLY CELL package, http://www.clcbio.com/products/clc-assembly-cell/) before undertaking sequence assembly. Second, the contigs were assembled using CLC de novo assembler with the following optimized parameters: bubble size of 98, minimum contig length of 200, mismatch cost of two, deletion and insertion costs of three, length fraction of 0.9, and similarity fraction of 0.8. Third, all the contigs were aligned to the reference chloroplast genome of *Machilus yunnanensis* (NC028073) using BLAST (http://blast.ncbi.nlm.nih.gov/), and aligned contigs (≥90% similarity and query coverage) were ordered according to the reference chloroplast genome. Then, contigs were aligned with the reference genome to construct the draft chloroplast genome of each species in Geneious 9.0.5 software (http://www.geneious.com). Finally, clean reads were re-mapped to the draft cp genomes of two *Phoebe* species, and the mapping ratio were 3.41% for *P. chekiangensis* and 2.15% for *P. bournei*, and average coverage depth were 578.2 and 367.1 for *P. chekiangensis* and *P. bournei*, respectively. Using the Dual Organellar Genome Annotator (DOGMA) program (Wyman et al. 2004), the two *Phoebe* chloroplast genomes were annotated. Protein-coding genes were identified by using the plastid/bacterial genetic code. Intron/exon boundaries were further determined using MAFFT v7 with those of the *M. yunnanensis* chloroplast genomes as a reference (Katoh and Standley 2013). Using the program tRNAscan-SEwith default settings (Schattner et al. 2005), tRNA boundaries were verified. The circular chloroplast genome maps of the *Phoebe* were drawn using the Organellar Genome DRAW (OGDRAW) software, with subsequent manual editing (Lohse et al. 2007).

### Characterization of repeat sequences and SSRs

REPuter was used to visualize both forward, palindrome, reverse and complement repeats, with a minimum repeat size of 30 bp and a sequence identity greater than 90% with hamming distance equal to 3 in *P. chekiangensis* and *P. bournei* (Kurtz and Schleiermacher 1999). The SSRs, which usually have a higher mutation rate, are easily genotyped using PCR, used as markers for phylogenetic analysis and in marker-assisted breeding when located on nuclear chromosomes. Microsatellites, or simple sequence repeats (SSRs) were detected using MISA perl script with thresholds of ten repeat units for mononucleotide SSRs, five repeat units for dinucleotide SSRs, four repeat units for trinucleotide SSRs, and three repeat units for tetra-, penta-, and hexanucleotide SSRs (Thiel et al. 2003).

### Divergence hotspot identification

The four complete chloroplast genome sequences (*P. chekiangensis*, *P. bournei*, *P. sheareri*, and *P. sheareri* var. *oineiensis*) were aligned,using the chloroplast genome of *M. yunnanensis* as a reference by mVISTA program (Frazer et al. 2004). Default parameters were utilized to align the chloroplast genomes in Shuffle-LAGAN mode and a sequence conservation profile was visualized in an mVISTA plot. To discover the divergence hotspot regions in *Phoebe*, protein coding gene, intron, and intergenic spacer region were evaluated with DnaSP 5.10 (Librado and Rozas 2009). All the regions were sequentially extracted under the following criteria: (1) total number of mutation (Eta) >0; (2) an aligned length >200 bp. Any large structural events, such as gene order rearrangements and IR expansions/contractions, were recorded.

## Results

### Chloroplast genomes organization of *P. chekiangensis* and *P. bournei*

**Fig. 1:**
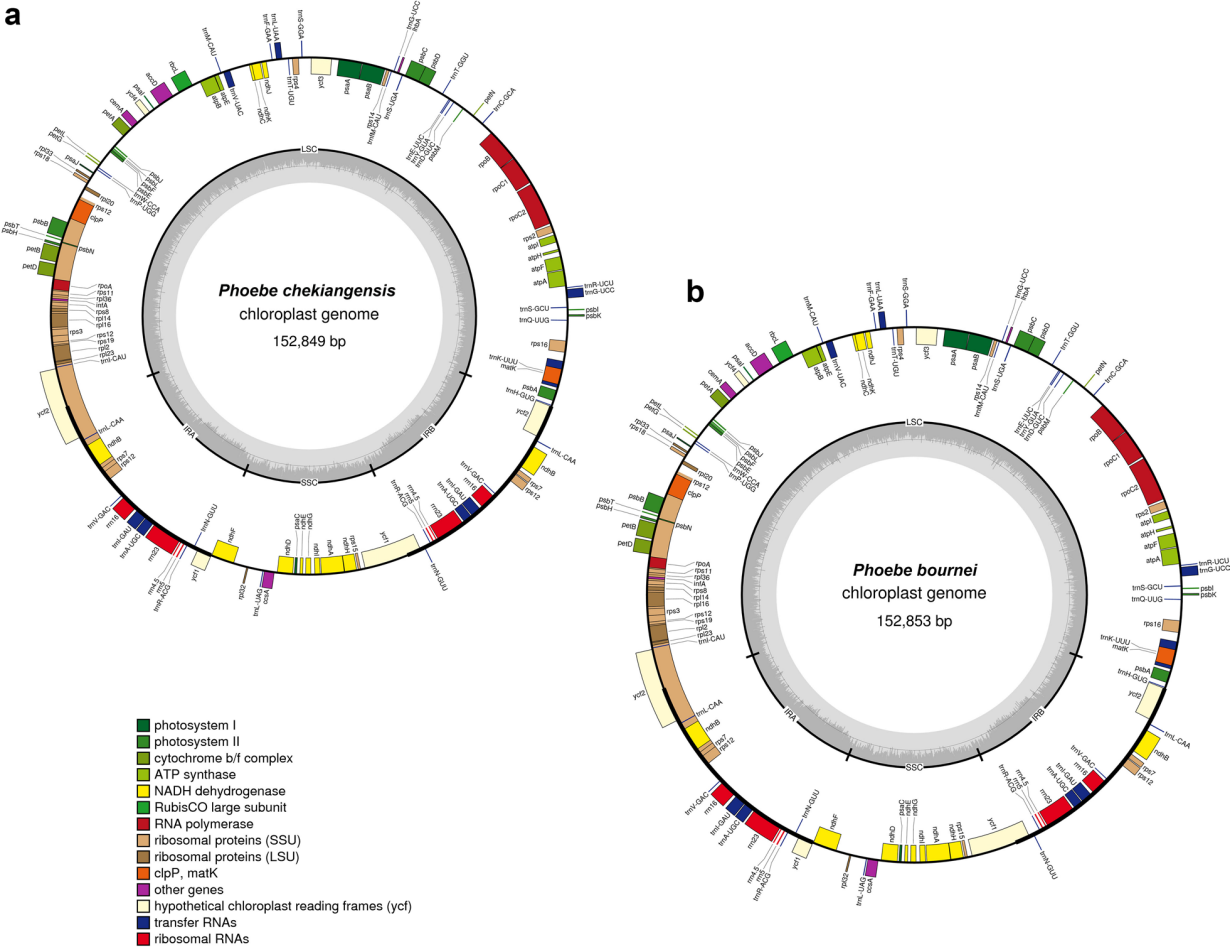
Chloroplast genome of *Phoebe chekiangensis* and *Phoebe bournei*. **a** *Phoebe chekiangensis*, **b** *Phoebe bournei*. Genes shown on the outside of the *circle* are transcribed clockwise, and genes inside are transcribed counter-clockwise. Large single copy (LSC), small single copy (SSC), and inverted repeats (IRa, IRb) are indicated. Genes belonging to different functional groups are* color-coded*. The *darker gray* in the* inner* corresponds to GC content, and the *lighter gray* corresponds to AT content

For these two *Phoebe* species, just three contigs which were found to be significantly homologous to the reference genome were combined to generate each chloroplast genome, with no gaps or missing nucleotides (Ns) found. The total genome size of *P. chekiangensis*, with a length of 152,849 bp (deposited in GenBank, Accession No. KY346511), was in close proximity to those of other *Phoebe* species and only 4, 6, and 27 bp smaller than that of *P. bournei* (152,853 bp, deposited in GenBank, Accession No. KY346512), *P. sheareri* var. *oineiensis* (152,855 bp, GenBank Accession No. KX437772), and *P. sheareri* (152,876 bp, GenBank Accession No. KX437773), respectively. The size of complete chloroplast genome of the four *Phoebe* species was within the range of angiosperms (Yang et al. 2010). Similar to the vast majority of angiosperms, showing a typical quadripartite structure, the two *Phoebe* chloroplast genomes consist of a pair of inverted repeats (IRs) of 18,927 bp in *P. chekiangensis* and 18,928 bp in *P.bournei*, a large single copy (LSC) region of 93,772 bp in *P. chekiangensis* and 93,777 bp in *P. bournei* and a small single copy (SSC) region of 20,775 bp in *P. chekiangensis* and 20,774 bp in *P. bournei* (Fig. 1; Table 1). Protein-coding regions accounted for 46.23% of the whole genome, while tRNA and rRNA regions accounted for 1.79 and 5.91%, respectively, and the remaining 46.07% was non-coding regions (Table 1). The overall GC content was 39.1%, whereas the GC content in the LSC, SSC and IR regions were 38.0, 34.4, and 44.4%, respectively, indicating identical levels among the two *Phoebe* chloroplast genomes.

**Table 1:** The basic characteristics of the sequencing data for *P. chekiangensis* and *P. bournei*

Characteristics	*P. chekiangensis*	*P. bournei*
Clean reads	20,777,674	20,787,108
Average read length	125	125
Total cpDNA size (bp)	152,849	152,853
Length of large single copy (LSC) region	93,772	93,777
Length of inverted repeat (IR) region	18,927	18,928
Length of small single copy (SSC) region	20,075	20,074
Total CDS length	70,668	70,668
Total tRNA length	2740	2740
Total rRNA length	9038	9038
Total GC content (%)	39.1	39.1
LSC	38.0	38.0
IR	44.4	44.4
SSC	34.4	34.4
Total number of genes	113	113
Protein-coding genes	79	79
rRNAs genes	4	4
tRNAs genes	30	30

### SSR and repeat sequences analysis

**Fig. 2:**
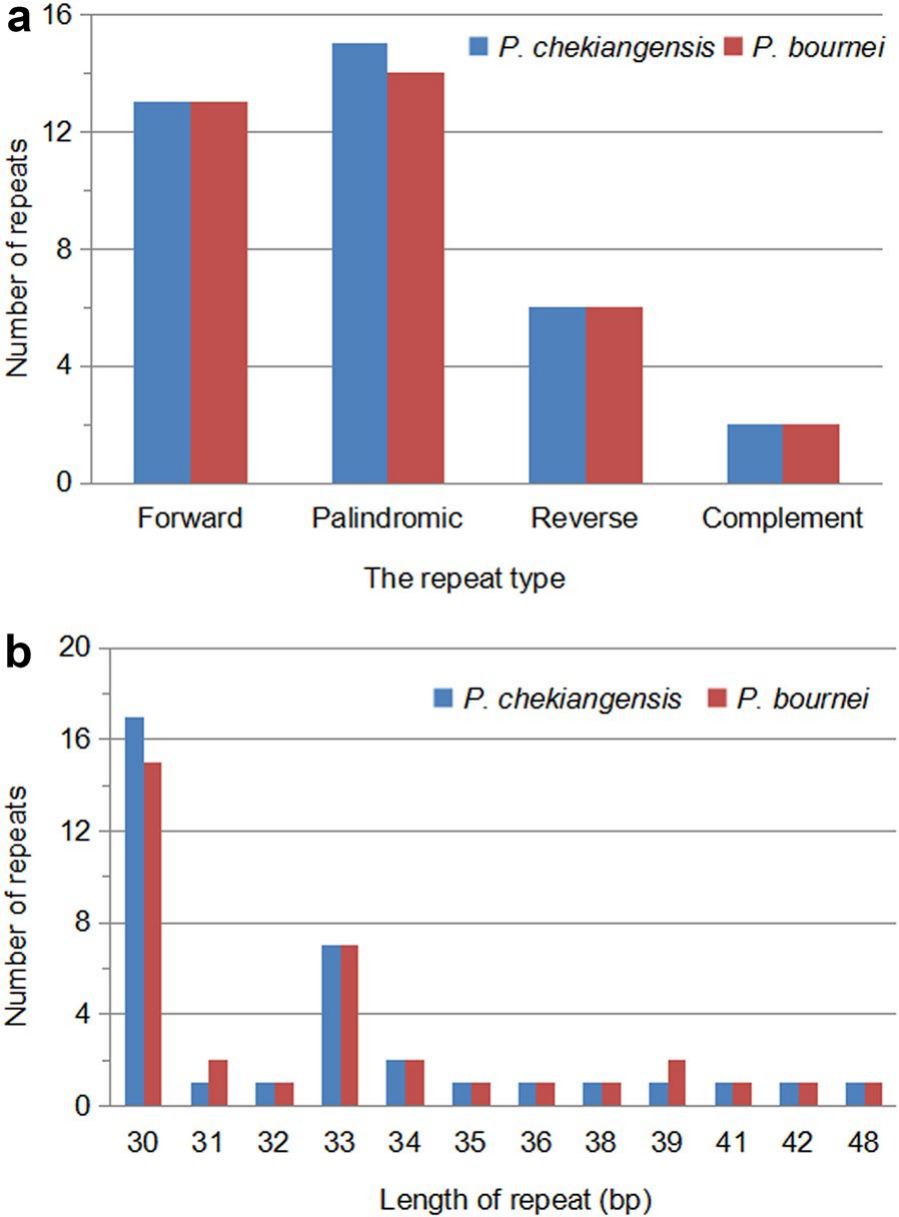
Analysis of repeated sequences in *P. chekiangensis* and *P. bournei* chloroplast genomes. **a** Frequency of repeats by length; **b** frequency of repeat types

Repeat sequences played a vital role in phylogenetic analysis and genome rearrangement (Nie et al. 2012). We used REPuter to analyze the repeat sequence of *Phoebe* cp genomes and found forward repeats, palindrome repeats and reverse repeats of at least 30 bp per repeat unit with a sequence identity of ≥90%. *P. chekiangensis* contained 36 repeats comprising of 13 forward repeats, 15 palindromic repeats, 6 reverse repeats and 2 complement repeats. The only quantity difference in repeat type between *P. chekiangensis* and *P. bournei* was that the latter contained 14 palindromic repeats, one less than the former (Fig. 2a; Additional file 1). The quantity of repeats with 30–40 bp length was 33 and 32 for *P. chekiangensis* and *P. bournei*, respectively. In addition, *P. chekiangensis* and *P. bournei* both contained one repeat with 41, 42, and 48 bp length (Fig. 2b; Additional file 1).

**Fig. 3:**
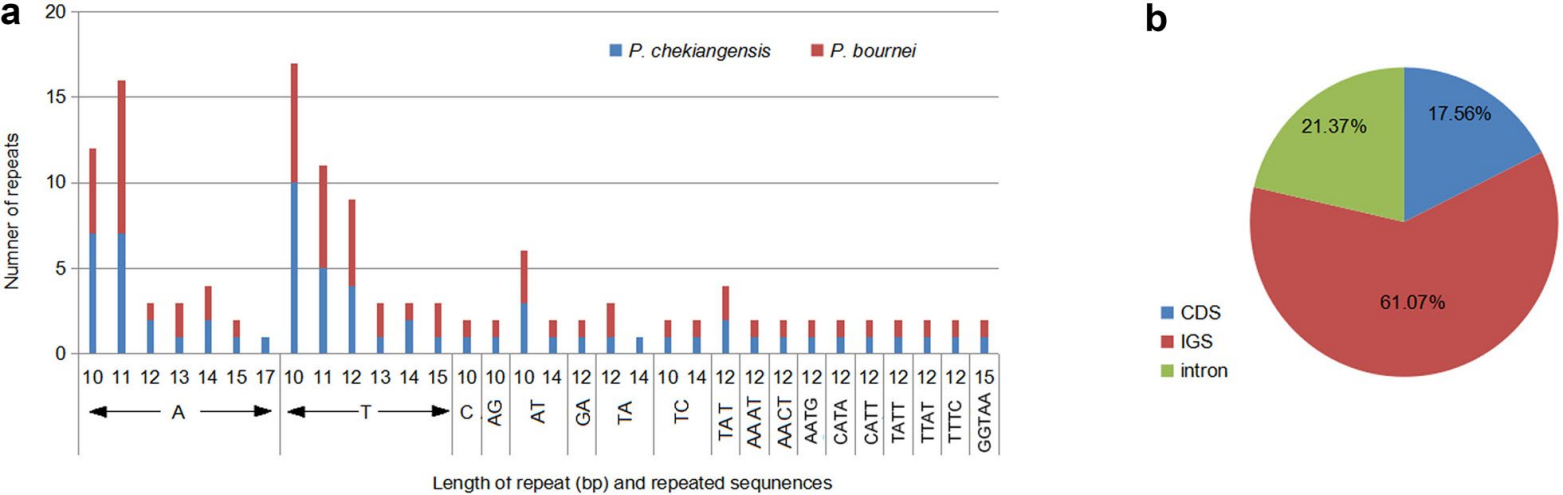
Simple sequence repeats (SSRs) in the two *Pheobe* chloroplast genomes. **a** Numbers of SSRs by length; **b** distribution of SSR loci. *IGS* intergenic spacer region

With MISA analysis, *Phoebe* chloroplast genome was found to contain 66 (*P. chekiangensis*) and 65 (*P. bournei*) SSRs longer than 10 bp, of which 49 SSRs were the same for the two chloroplast genomes (similar repeat units located in similar genomic regions) (Fig. 3a; Additional file 2). Among the total 131 SSRs, most loci were located in intergenic spacer (IGS) regions (61.07%), followed by introns (23.37%) and CDS (17.56%) (Fig. 3b). We observed that 12 different SSRs were located in five protein-coding genes [*ycf1* (×5), *cemA*(×2), *rpoC2* (×2), *ycf2* (×2), and *matK*] of the two *Phoebe* chloroplast genomes.

### Inverted repeats (IRs) contraction and expansion of four *Phoebe* species

**Fig. 4:**
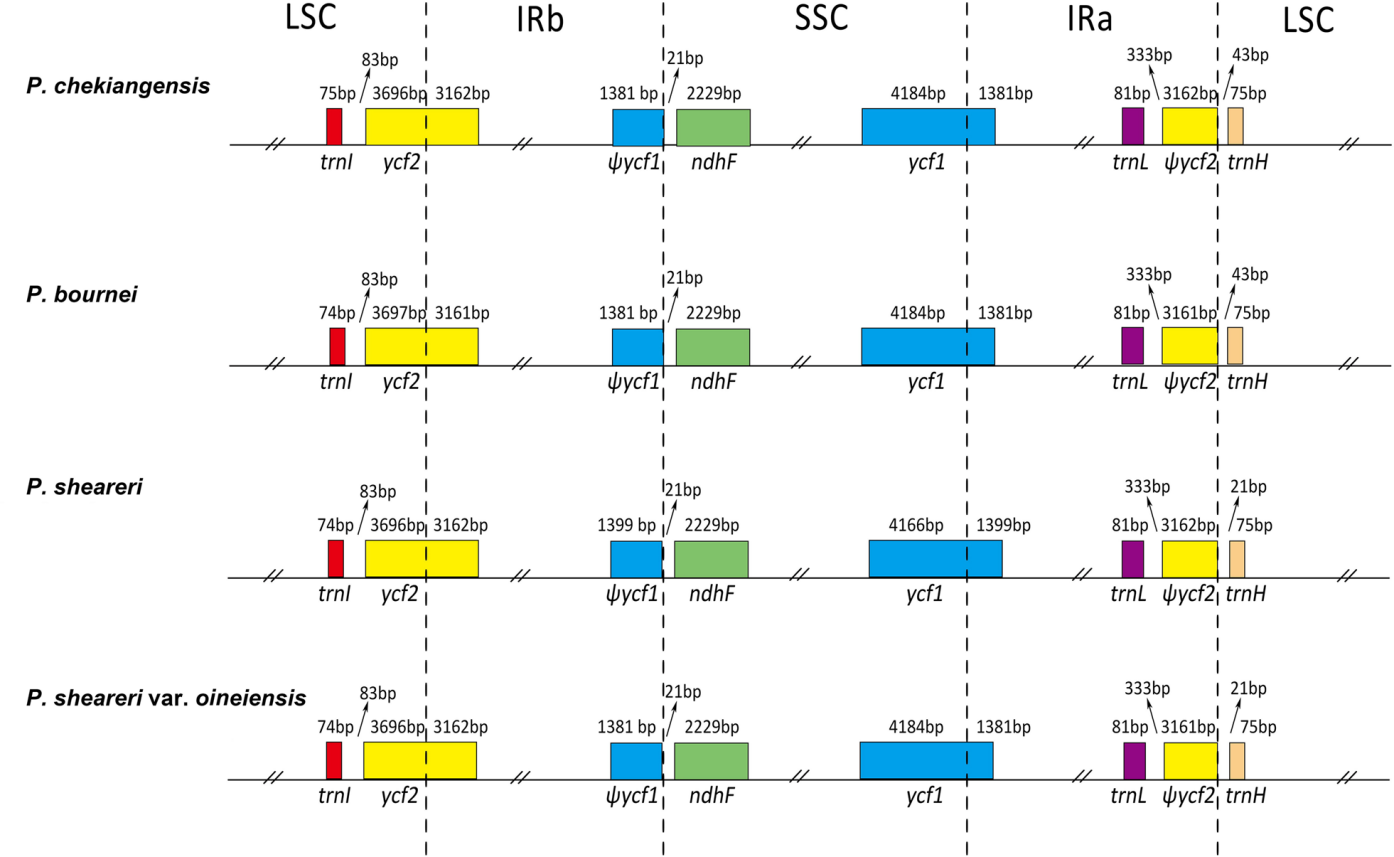
Comparison of LSC, IR, and SSC junction positions among chloroplast genomes of *P. chekiangensis*, *P. bournei*, *P. sheareri*, and *P. sheareri*var. *oineiensis*

The IR region expanded into the *ycf2* gene, creating a pseudogene fragment *ψycf2* at the IRa/LSC border with length of 3,161 bp (*P. bournei* and *P. sheareri* var. *oineiensis*) and 3,162 bp (*P. chekiangensi* and *P. sheareri*). The *ycf1* gene crossed the SSC/IRa region and the pseudogene fragment ψ*ycf1* was located at the IRb region with 1381 and 1399 bp. The *trnH* was the unique gene with large difference among *Phoebe* species. The *trnH* genes of *P. sheareri* and *P. sheareri* var. *oineiensis* were separated by 21 bp at the IRa/LSC border, whereas those of *P. bournei* and *P. chekiangensis* were separated by 43 bp (Fig. 4).

### Divergence sequence hotspots in *Phoebe* species

**Fig. 5:**
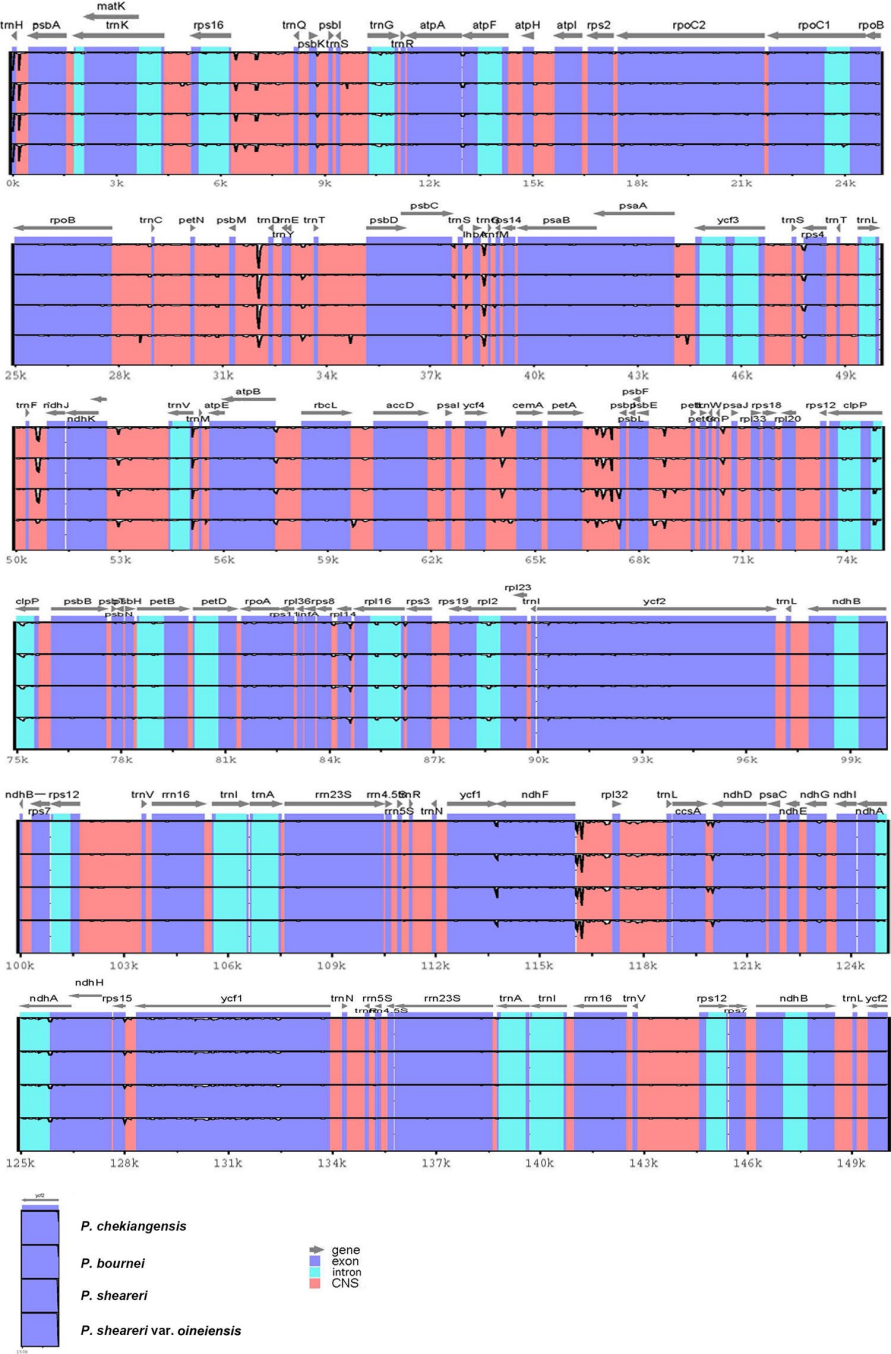
Sequence identity plots among chloroplast genomes of *P. chekiangensis*, *P. bournei*, *P. sheareri*, and *P. sheareri* var. *oineiensis*, with *Machilus yunnanensis* as a reference. Annotated genes are displayed along the* top*. The *vertical scale* represents the percent identity between 50 and 100%. Genome regions are *color* coded as exon, intron, and conserved non-coding sequences (CDS)

The overall sequence identity of the four *Phoebe* chloroplast genomes was compared and plotted using the mVISTA program (Frazer et al. 2004), with the annotation of *M. yunnanensis* as a reference to elucidate the level of sequence divergence (Fig. 5). Being largely consistent with recent studies (Yao et al. 2015; Zhang et al. 2016), most of the sequence variations were found to be located in the LSC and SSC regions, while the IR regions exhibited comparatively lower sequence diversity. The lower sequence divergence observed in the IRs rather than LSC and SSC regions for *Phoebe* species is likely due to copy correction between IR sequences by gene conversion (Khakhlova and Bock 2006).

**Fig. 6:**
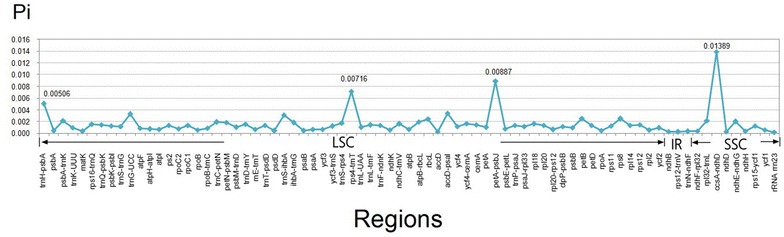
The nucleotide diversity (Pi) values were compared among four *Phoebe* species

Seventy-seven regions (30 coding regions, 38 intergenic spacers, eight introns, and one rRNA) with more than 200 bp in length were eventually identified. Of these 77 regions, nucleotide diversity (*Pi*) ranged from 0.00018 (*rrn23*) to 0.01389 (*ccsA*-*ndhD*) among four *Phoebe* species (Fig. 6; Additional file 3). As found in most angiosperms (Choi et al. 2016), sequence divergence in intergenic regions was higher than that in genic regions of these four *Phoebe* chloroplast genomes. The mean value of *Pi* in non-coding regions was 0.221%, which was nearly more than twice as much as that (0.123% on average) in the coding regions. Intergenic regions with a percentage of *Pi* exceeding 0.5% were *trnH*-*psbA* (0.506%), *rps4*-*trnT* (0.716%), *petA*-*psbJ* (0.887%), and *ccsA*-*ndhD* (1.389%). However, the highest proportion of variability in genic regions was 0.251% (*rps8*) (Fig. 6; Additional file 3).

## Discussion

Results from this study showed the GC content of *P. chekiangensis* and *P. bournei* chloroplast genomes is close to that reported in other Lauraceae chloroplast genomes (Song et al. 2015, 2016). Although the GC percentage in the IR regions (44.4%) of the two *Pheobe* was higher than that of *Nicotiana otophora* (43%) (Asaf et al. 2016), the presence of rRNA in *Pheobe* (four) was lower than that of *N. otophora* (eight). The results in this study were incompatible to previous report which suggested that a high GC percentage in the IR regions could be due to the presence of rRNA (Qian et al. 2013). Similarly, 112 different genes, including 78 protein-coding genes (47 genes encoding photosynthesis-related proteins, four DNA dependent RNA polymerases, 20 ribosomal proteins, one translation initiation factor, four genes encoding other proteins, and two genes of unknown function), 30 tRNA genes, and four rRNA genes were annotated in *P. chekiangensis* and *P. bournei* chloroplast genomes (Fig. 1; Additional file 4). The pattern of protein coding genes was similar to that of *Persea americana* (Song et al. 2016). Among all the protein-coding genes, ten genes possessed a single intron, two genes (*ycf3* and *clpP*) contain two introns, whereas six tRNA genes contain a single intron (Additional file 4).

Overall, a total of 71 repeats, including 36.6% forward repeats (26), 40.8% palindromic repeats (29), 16.9% reverse repeats (12), and 5.7% complement repeats (4), were detected in *P. chekiangensis* and *P. bournei* chloroplast genomes. About 61.07% of these repeats were distributed in intergenic spacer. The result was comparable to chloroplast genomes of most angiosperm plant (Uthaipaisanwong et al. 2012; Yao et al. 2015). Previous studies suggested that the presence of these repeats indicates that the region is a crucial hotspot for genome reconfiguration (Gao et al. 2009). One 30 bp forward repeat occurred in the *ndhC*-*trnV* intergenic spacer and one 30 bp palindromic repeat in *Ψycf1* were unique to *P. chekiangensis*. In contrast, one 31 bp forward repeat occurred in the *ndhC*-*trnV* intergenic spacer were only contained by *P. bournei* (Additional file 1). Apart from the above three repeats, the others were shared between two *Phoebe* species. SSRs in the chloroplast with high polymorphism in copy numbers have been recognized as one of the main sources of molecular markers, and extensively used for population genetics and phylogenetic investigation (Pauwels et al. 2012; Zhang et al. 2012a, b; Zhao et al. 2015).

Among 131 SSRs longer than 10 bp, almost all mononucleotide was composed of A/T (97.7%), and majority of dinucleotides was composed of AT (60.0%). The AT richness in SSRs of the two *Pheobe* genome was similar to previous reports suggesting that SSRs found in the chloroplast genome were generally composed of polythymine (T) or polyadenine (A) repeats, and infrequently contained tandem cytosine (C) and guanine (G) repeats (Kuang et al. 2011; Qian et al. 2013; Chen et al. 2015). The SSRs identified in the two *Phoebe* species might be used in future population genetic studies as well as similar studies of other species, such as *Panax ginseng* (Kim and Lee 2004), *Cucumis sativus* (Kim et al. 2006), *Vigna radiate* (Tangphatsornruang et al. 2010), and *Pyrus pyrifolia* (Terakami et al. 2012).

Based on the analysis of inverted repeats contraction and expansion, a conclusion could be drawn that chloroplast genomes of four *Phoebe* species exhibited comparatively little difference at the IR/LSC and IR/SSC boundary regions, which was similar in Veroniceae (Choi et al. 2016). The genetic divergence within Lauraceaeis surprisingly low (Rohwer 2000; Song et al. 2016). The mean nucleotide diversity of the complete chloroplast genome of the four *Phoebe* species was only 0.162%, lower than that of two *Panax* species (0.40%) (Dong et al. 2014), three *Veroniceae* species (0.40%) (Choi et al. 2016), and nine *Gossypium* species (0.62%) (Xu et al. 2012), and extremely lower than that of five *Epimedium* species (3.97%) (Zhang et al. 2016). Molecular markers with nucleotide diversity over 1.5% have been reported before as highly variable regions and could be used to promote the further phylogenetic analysis and species identification in other seed plants (Särkinen and George 2013; Korotkova et al. 2014; Huang et al. 2014). However, due to the relative conservative of chloroplast genomes of *Phoebe* species, the quantity of the regions with nucleotide diversity exceeding 0.5% was only four, whereas in *Nicotiana* (Asaf et al. 2016), the quantity of the regions with nucleotide diversity over 0.7% was 15, and in *Machilus* (Song et al. 2015), the quantity of the regions with nucleotide diversity over 0.8% was 7. In this study, the highest nucleotide diversity was 1.389% (*ccsA*-*ndhD*), followed by 0.887% (*petA*-*psbJ*), 0.716% (*rps4*-*trnT*), and 0.506% (*trnH*-*psbA*). Therefore, only the above four regions with nucleotide diversity higher than 0.5% probably had the potential use for phylogeographic analyses and plant identification of *Phoebe* species.

## Conclusions

The 77 repeat sequences were identified in the *Phoebe* chloroplast genomes. The analysis showed the extremely conserved structure of chloroplast genomes, the mean nucleotide diversity was found to be 0.162% for 77 regions, suggesting an extraordinarily low level of sequence divergence. Four highest divergent regions (*trnH*-*psbA, rps14*-*trnT, petA*-*psbJ, ccsA*-*ndhD*) were also identified, which might be useful in species identification and phylogenetic studies. Overall, this study will facilitate our understanding of population genetics, phylogenetic relationship and plant evolution of *Phoebe* species.

## Additional files

**Additional file 1: Table S1.** Analyses of repeat sequences in the two *Phoebe* chloroplast genomes.

**Additional file 2: Table S2.** The simple sequence repeats in *P. chekiangensis* and *P. bournei.*

**Additional file 3: Table S3.** Nucleotide diversity (*Pi*) values and total number of mutation (Eta) in *Phoebe.*

**Additional file 4: Table S4.** Genes, separated by category, encoded by *P. chekiangensis* and *P. bournei*plastomes.
