# Supplementary material for: Complete chloroplast genome sequences of two endangered Phoebe (Lauraceae) species
Source: Bot Stud. 2017 Sep 13;58:37. doi: 10.1186/s40529-017-0192-8 (PMC5597560; doi:10.1186/s40529-017-0192-8)
Supplement: Supplementary file 1 — Additional file 1: Table S1. Analyses of repeat sequences in the two Phoebe chloroplast genomes. [file 40529_2017_192_MOESM1_ESM.docx]

| Table S1. Analyses of repeat sequences in the two *Phoebe* chloroplast genomes | | | | | | | |
| --- | --- | --- | --- | --- | --- | --- | --- |
| *P. chekiangensi* | | | Location | *P. bournei* | | | Location |
| Repeat  types | Repeat  length | Start  position |  | Repeat  types | Repeat  length | start  position |  |
| F | 42 | 67273 | IGS petA-psbJ | F | 42 | 67275 | IGS petA-psbJ |
| F | 41 | 40988 | CDS psaB | F | 41 | 40988 | CDS psaB |
| F | 39 | 45902 | intron ycf3 | F | 39 | 45902 | intron ycf3 |
| F | 36 | 132745 | CDS ycf1 | F | 36 | 132750 | CDS ycf1 |
| F | 34 | 87893 | IGS rps19-rpl2 | F | 34 | 87898 | IGS rps19-rpl2 |
| F | 33 | 95100 | CDS ycf2 | F | 33 | 95104 | CDS ycf2 |
| F | 33 | 151464 | Ψycf2 | F | 33 | 151469 | Ψycf2 |
| F | 33 | 97231 | IGS ycf2-trnL | F | 33 | 97235 | IGS ycf2-trnL |
| F | 32 | 9450 | trnS-GCU | F | 32 | 9443 | trnS-GCU |
| F | 30 | 11158 | trnG-UCC | F | 31 | 53069 | IGS ndhC-trnV |
| F | 30 | 33305 | IGS trnE-trnT | F | 30 | 11162 | trnG-UCC |
| F | 30 | 45914 | intron ycf3 | F | 30 | 33309 | IGS trnE-trnT |
| F | 30 | 53069 | IGS ndhC-trnV | F | 30 | 45914 | intron ycf3 |
| P | 48 | 77939 | IGS psbT-psbN | P | 48 | 77942 | IGS psbT-psbN |
| P | 39 | 45902 | intron ycf3 | P | 39 | 45902 | intron ycf3 |
| P | 38 | 48792 | IGS rps4-trnT | P | 38 | 48792 | IGS rps4-trnT |
| P | 34 | 87893 | IGS rps19-rpl2 | P | 34 | 87898 | IGS rps19-rpl2 |
| P | 33 | 95100 | CDS ycf2 | P | 33 | 95104 | CDS ycf2 |
| P | 33 | 95124 | CDS ycf2 | P | 33 | 95128 | CDS ycf2 |
| P | 33 | 97231 | IGS ycf2-trnL | P | 33 | 97235 | IGS ycf2-trnL |
| P | 33 | 149357 | IGS trnL-ycf2 | P | 33 | 149362 | IGS trnL-ycf2 |
| P | 31 | 117681 | IGS rpl32-trnL | P | 31 | 117686 | IGS rpl32-trnL |
| P | 30 | 67482 | IGS petA-psbJ | P | 30 | 67484 | IGS petA-psbJ |
| P | 30 | 9452 | trnS-GCU | P | 30 | 9445 | trnS-GCU |
| P | 30 | 119631 | CDS ccsA | P | 30 | 119636 | CDS ccsA |
| P | 30 | 37857 | trnS-UGA | P | 30 | 37859 | trnS-UGA |
| P | 30 | 45914 | intron ycf3 | P | 30 | 45914 | intron ycf3 |
| P | 30 | 113817 | Ψycf1 | R | 35 | 116416 | IGS ndhF-rpl32 |
| R | 35 | 116410 | IGS ndhF-rpl32 | R | 30 | 102162 | IGS rps12-trnV |
| R | 30 | 102158 | IGS rps12-trnV | R | 30 | 102166 | IGS rps12-trnV |
| R | 30 | 102162 | IGS rps12-trnV | R | 30 | 144434 | IGS trnV-rps12 |
| R | 30 | 144429 | IGS trnV-rps12 | R | 30 | 144438 | IGS trnV-rps12 |
| R | 30 | 144433 | IGS trnV-rps12 | R | 30 | 48535 | IGS rps4-trnT |
| R | 30 | 48535 | IGS rps4-trnT | C | 30 | 102162 | IGS rps12-trnV |
| C | 30 | 102158 | IGS rps12-trnV | C | 30 | 102166 | IGS rps12-trnV |
| C | 30 | 102162 | IGS rps12-trnV |  |  |  |  |
| F: Forward; R: Reverse; P: Palindrome C: Complement | | | | | | | |
