# Supplementary material for: Complete chloroplast genome sequences of two endangered Phoebe (Lauraceae) species
Source: Bot Stud. 2017 Sep 13;58:37. doi: 10.1186/s40529-017-0192-8 (PMC5597560; doi:10.1186/s40529-017-0192-8)
Supplement: Supplementary file 2 — Additional file 2: Table S2. The simple sequence repeats in P. chekiangensis and P. bournei. [file 40529_2017_192_MOESM2_ESM.docx]

| Table S2. The simple sequence repeats in *P. chekiangensis* and *P. bournei* | | | | | | | | | |
| --- | --- | --- | --- | --- | --- | --- | --- | --- | --- |
| *P. chekiangensis* | | | | | *P. bournei* | | | | |
| Repeat  unit | No.  repeat  unit | start | end | location | Repeat  unit | No.  repeat  unit | start | end | location |
| A | 10 | 8863 | 8872 | IGS psbK psbI | A | 10 | 8856 | 8865 | IGS psbK psbI |
|  |  | 31945 | 31954 | IGS psbM trnD |  |  | 15159 | 15168 | IGS atpH atpI |
|  |  | 38103 | 38112 | IGS trnS ihbA |  |  | 38105 | 38114 | IGS trnS ihbA |
|  |  | 53940 | 53949 | IGS ndhK trnV |  |  | 92105 | 92114 | CDS ycf2 |
|  |  | 92101 | 92110 | CDS ycf2 |  |  | 113375 | 113384 | CDS ycf1 |
|  |  | 113371 | 113380 | Ψycf1 |  | 11 | 5615 | 5625 | intron rps16 |
|  |  | 117085 | 117094 | IGS ndhF rpl32 |  |  | 14160 | 14170 | intron atpF |
|  | 11 | 14158 | 14168 | intron atpF |  |  | 31948 | 31958 | IGS psbM trnD |
|  |  | 15157 | 15167 | IGS atpH atpI |  |  | 32682 | 32692 | IGS trnD trnY |
|  |  | 32678 | 32688 | IGS trnD trnY |  |  | 49130 | 49140 | IGS trnT trnL |
|  |  | 49130 | 49140 | IGS trnT trnL |  |  | 66997 | 67007 | IGS petA psbJ |
|  |  | 66995 | 67005 | IGS petA psbJ |  |  | 116279 | 116289 | IGS ndhF rpl32 |
|  |  | 117698 | 117708 | IGS rpl32 trnL |  |  | 117703 | 117713 | IGS rpl32 trnL |
|  |  | 131956 | 131966 | CDS ycf1 |  |  | 131961 | 131971 | CDS ycf1 |
|  | 12 | 5621 | 5632 | intron rps16 |  | 12 | 74924 | 74935 | intron clpP |
|  |  | 74922 | 74933 | intron clpP |  | 13 | 13056 | 13068 | IGS atpA atpF |
|  | 13 | 72826 | 72838 | IGS rpl20 rps12 |  |  | 72828 | 72840 | IGS rpl20 rps12 |
|  | 14 | 13052 | 13065 | IGS atpA atpF |  | 14 | 243 | 256 | IGS trnH-psbA |
|  |  | 118094 | 118107 | IGS rpl32 trnL |  |  | 118099 | 118112 | IGS rpl32 trnL |
|  | 15 | 243 | 257 | IGS trnH-psbA |  | 15 | 38949 | 38963 | IGS trnG trnfM |
|  | 17 | 38947 | 38963 | IGS trnG trnfM | T | 10 | 15647 | 15656 | IGS atpH atpI |
| T | 10 | 15646 | 15655 | IGS atpH atpI |  |  | 24021 | 24030 | intron rpoC1 |
|  |  | 17425 | 17434 | IGS rps2 rpoC2 |  |  | 29855 | 29864 | IGS trnC petN |
|  |  | 29852 | 29861 | IGS trnC petN |  |  | 31423 | 31432 | IGS psbM trnD |
|  |  | 31420 | 31429 | IGS psbM trnD |  |  | 84707 | 84716 | IGS rpl14 rpl16 |
|  |  | 66584 | 66593 | IGS petA psbJ |  |  | 129749 | 129758 | CDS ycf1 |
|  |  | 75238 | 75247 | intron clpP |  |  | 133247 | 133256 | CDS ycf1 |
|  |  | 84703 | 84712 | IGS rpl14 rpl16 |  | 11 | 7977 | 7987 | IGS rps16 trnQ |
|  |  | 87873 | 87882 | IGS rps19 rpl2 |  |  | 17426 | 17436 | IGS rps2 rpoC2 |
|  |  | 129744 | 129753 | CDS ycf1 |  |  | 19602 | 19612 | CDS rpoC2 |
|  |  | 133242 | 133251 | CDS ycf1 |  |  | 45043 | 45053 | intron ycf3 |
|  | 11 | 7984 | 7994 | IGS rps16 trnQ |  |  | 66585 | 66595 | IGS petA psbJ |
|  |  | 10595 | 10605 | tRNA trnG |  |  | 75240 | 75250 | intron clpP |
|  |  | 13751 | 13761 | intron atpF |  | 12 | 4630 | 4641 | IGS matK rps16 |
|  |  | 19600 | 19610 | CDS rpoC2 |  |  | 5987 | 5998 | intron rps16 |
|  |  | 45043 | 45053 | intron ycf3 |  |  | 33350 | 33361 | IGS trnE trnT |
|  | 12 | 4631 | 4642 | IGS matK rps16 |  |  | 55524 | 55535 | IGS trnM atpE |
|  |  | 5994 | 6005 | intron rps16 |  |  | 74245 | 74256 | intron clpP |
|  |  | 55524 | 55535 | IGS trnM atpE |  | 13 | 10597 | 10609 | tRNA trnG |
|  |  | 74243 | 74254 | intron clpP |  |  | 72147 | 72159 | IGS rps18 rpl20 |
|  | 13 | 72145 | 72157 | IGS rps18 rpl20 |  | 14 | 86273 | 86286 | IGS rpl16 rps3 |
|  | 14 | 33346 | 33359 | IGS trnE trnT |  | 15 | 13754 | 13768 | intron atpF |
|  |  | 86269 | 86282 | IGS rpl16 rps3 |  |  | 44223 | 44237 | IGS psaA ycf3 |
|  | 15 | 44223 | 44237 | IGS psaA ycf3 | C | 10 | 5199 | 5208 | IGS matK rps16 |
| C | 10 | 5205 | 5214 | IGS matK rps16 | AG | 5 | 3099 | 3108 | CDS matK |
| AG | 5 | 3100 | 3109 | CDS matK | AT | 5 | 20975 | 20984 | CDS rpoC2 |
| AT | 5 | 20973 | 20982 | CDS rpoC2 |  |  | 28035 | 28044 | IGS rpoB trnC |
|  |  | 28032 | 28041 | IGS rpoB trnC |  |  | 31089 | 31098 | IGS petN psbM |
|  |  | 31086 | 31095 | IGS petN psbM |  | 7 | 116911 | 116924 | IGS ndhF rpl32 |
|  | 7 | 116905 | 116918 | IGS ndhF rpl32 | GA | 6 | 93304 | 93315 | CDS ycf2 |
| GA | 6 | 93300 | 93311 | CDS ycf2 | TA | 6 | 34752 | 34765 | IGS trnT psbD |
| TA | 6 | 88442 | 88453 | intron rpl2 |  |  | 88447 | 88458 | intron rpl2 |
|  | 7 | 34750 | 34763 | IGS trnT psbD | TC | 5 | 64581 | 64590 | CDS cemA |
| TC | 5 | 64580 | 64589 | CDS cemA |  | 7 | 87031 | 87044 | IGS rps3 rps19 |
|  | 7 | 87027 | 87040 | IGS rps3 rps19 | TAT | 4 | 68726 | 68737 | IGS psbE petL |
| TAT | 4 | 68724 | 68735 | IGS psbE petL |  |  | 74470 | 74481 | intron clpP |
|  |  | 74468 | 74479 | intron clpP | AAAT | 3 | 144435 | 144446 | IGS trnV rps12 |
| AAAT | 3 | 144430 | 144441 | IGS trnV rps12 | AACT | 3 | 48928 | 48939 | IGS trnT trnL |
| AACT | 3 | 48928 | 48939 | IGS trnT trnL | AATG | 3 | 65191 | 65202 | CDS cemA |
| AATG | 3 | 65190 | 65201 | CDS cemA | CATA | 3 | 32480 | 32491 | IGS trnD trnY |
| CATA | 3 | 32476 | 32487 | IGS trnD trnY | CATT | 3 | 130385 | 130396 | CDS ycf1 |
| CATT | 3 | 130380 | 130391 | CDS ycf1 | TAAA | 3 | 46565 | 46576 | intron ycf3 |
| TAAA | 3 | 46565 | 46576 | intron ycf3 | TTTA | 3 | 102182 | 102193 | intron rps12 |
| TTTA | 3 | 102178 | 102189 | intron rps12 | TTTC | 3 | 81546 | 81557 | IGS psbN psbH |
| TTTC | 3 | 81543 | 81554 | IGS psbN psbH | GGTAA | 3 | 38813 | 38827 | IGS trnG trnfM |
| GGTAA | 3 | 38811 | 38825 | IGS trnG trnfM |  |  |  |  |  |
