# Supplementary material for: Complete chloroplast genome sequences of two endangered Phoebe (Lauraceae) species
Source: Bot Stud. 2017 Sep 13;58:37. doi: 10.1186/s40529-017-0192-8 (PMC5597560; doi:10.1186/s40529-017-0192-8)
Supplement: Supplementary file 3 — Additional file 3: Table S3. Nucleotide diversity (Pi) values and total number of mutation (Eta) in Phoebe. [file 40529_2017_192_MOESM3_ESM.docx]

| Table S3. Nucleotide diversity (Pi) values and total number of mutation (Eta) in *Phoebe* | | |
| --- | --- | --- |
| region | Eta | Pi |
| IGS trnH-psbA | 4 | 0.00506 |
| CDS psbA | 1 | 0.00047 |
| IGS psbA-trnK | 1 | 0.00214 |
| intron trnK-UUU | 5 | 0.00099 |
| CDS matK | 1 | 0.00036 |
| IGS rps16-trnQ | 6 | 0.00158 |
| IGS trnQ-psbK | 1 | 0.00149 |
| IGS psbK-psbI | 1 | 0.00131 |
| IGS trnS-trnG | 2 | 0.00122 |
| intron trnG-UCC | 5 | 0.0033 |
| intron atpF | 1 | 0.00092 |
| IGS atpH-atpI | 1 | 0.00075 |
| CDS atpI | 1 | 0.00067 |
| CDS rps2 | 2 | 0.00141 |
| CDS rpoC2 | 6 | 0.00079 |
| intron rpoC1 | 2 | 0.00139 |
| CDS rpoB | 4 | 0.00063 |
| IGS rpoB-trnC | 4 | 0.00087 |
| IGS trnC-petN | 4 | 0.00191 |
| IGS petN-psbM | 4 | 0.00188 |
| IGS psbM-trnD | 2 | 0.00103 |
| IGS trnD-trnY | 1 | 0.0016 |
| IGS trnE-trnT | 1 | 0.00069 |
| IGS trnT-psdD | 4 | 0.00139 |
| CDS psdD | 1 | 0.00047 |
| IGS trnS-ihbA | 2 | 0.00312 |
| IGS ihbA-trnG | 1 | 0.00182 |
| CDS psaB | 2 | 0.00045 |
| CDS psaA | 3 | 0.00067 |
| intron ycf3 | 2 | 0.00068 |
| IGS ycf3-trnS | 2 | 0.00124 |
| IGS trnS-rps4 | 1 | 0.0018 |
| IGS rps4-trnT | 5 | 0.00716 |
| intron trnL-UAA | 1 | 0.00104 |
| IGS trnL-trnF | 1 | 0.00144 |
| IGS trnF-ndhK | 3 | 0.00136 |
| CDS ndhK | 1 | 0.00058 |
| IGS ndhC-trnV | 6 | 0.00162 |
| CDS atpB | 2 | 0.00067 |
| IGS atpB-rbcL | 3 | 0.00199 |
| CDS rbcL | 7 | 0.00245 |
| CDS accD | 1 | 0.00033 |
| IGS accD-psal | 4 | 0.00342 |
| CDS ycf4 | 1 | 0.0012 |
| IGS ycf4-cemA | 3 | 0.00166 |
| CDS cemA | 2 | 0.00145 |
| CDS petA | 2 | 0.00104 |
| IGS petA-psbJ | 16 | 0.00887 |
| IGS psbE-petL | 2 | 0.00078 |
| IGS trnP-psaJ | 1 | 0.00136 |
| IGS psaJ-rpl33 | 1 | 0.00117 |
| CDS rpl18 | 1 | 0.00163 |
| CDS rpl20 | 1 | 0.00141 |
| IGS rpl20-rps12 | 1 | 0.00066 |
| IGS clpP-psbB | 1 | 0.00117 |
| CDS psbB | 3 | 0.00098 |
| intron petB | 4 | 0.00254 |
| CDS petD | 1 | 0.00136 |
| CDS rpoA | 1 | 0.00049 |
| CDS rps11 | 1 | 0.00127 |
| CDS rps8 | 2 | 0.00251 |
| CDS rpl14 | 1 | 0.00136 |
| intron rps12 | 2 | 0.00147 |
| CDS rpl2 | 1 | 0.00061 |
| CDS ycf2 | 13 | 0.00095 |
| CDS ndhB | 1 | 0.00033 |
| IGS rps12-trnV | 1 | 0.00027 |
| IGS trnN-ndhF | 1 | 0.00037 |
| IGS ndhF-rpl32 | 1 | 0.0004 |
| IGS rpl32-trnL | 6 | 0.00218 |
| IGS ccsA-ndhD | 5 | 0.01389 |
| CDS ndhD | 1 | 0.00033 |
| IGS ndhE-ndhG | 1 | 0.00202 |
| CDS ndhH | 1 | 0.00042 |
| IGS rps15-ycf1 | 1 | 0.0013 |
| CDS ycf1 | 7 | 0.00063 |
| rRNA rrn23 | 1 | 0.00018 |
| * IGS: Intergenic spacer region; Eta: total number of mutation | | |
