# Supplementary material for: Complete chloroplast genome sequences of two endangered Phoebe (Lauraceae) species
Source: Bot Stud. 2017 Sep 13;58:37. doi: 10.1186/s40529-017-0192-8 (PMC5597560; doi:10.1186/s40529-017-0192-8)
Supplement: Supplementary file 4 — Additional file 4: Table S4. Genes, separated by category, encoded by P. chekiangensis and P. bourneiplastomes. [file 40529_2017_192_MOESM4_ESM.docx]

| Table S4. Genes, separated by category, encoded by *P. chekiangensis* and *P. bournei* plastomes | |
| --- | --- |
| Group of gene | Name of gene |
| Photosynthesis-related genes |  |
| Rubisco | *rbcL* |
| Photosystem I | *psaA, psaB, psaC, psaI, psaJ* |
| Assembly and stability of photosystem I | *ycf3*^b^*, ycf4* |
| Photosystem II | *psbA, psbB, psbC, psbD, psbE, psbF, psbH, psbI, ppsbJ, psbK, psbL, psbM, psbN, psbT, psbZ* |
| ATP synthase | *atpA, atpB, atpE, atpF*^a^*, atpH, atpI* |
| Cytochrome b/f complex | *petA, petB*^a^*, petD*^a^*, petG, petL, petN* |
| Cytochrome c synthesis | *ccsA* |
| NADPH dehydrogenase | *ndhA*^a^*, ndhB*^a^*, ndhC, ndhD, ndhE, ndhF, ndhG, ndhH, ndhI, ndhJ, ndhK* |
| Transcription- and translation-related genes |  |
| Transcription | *rpoA, rpoB, rpoC1*^a^*, rpoC2* |
| Ribosomal proteins | *rps2, rps3, rps4, rps7, rps8, rps11, rps12*^a^*, rps14, rps15, rps16*^a^*, rps18, rps19, rpl2*^a^*, rpl14, rpl16*^a^*, rpl20, rpl23, rpl32, rpl33, rpl36* |
| Translation initiation factor | *infA* |
| RNA genes |  |
| Ribosomal RNA | *rrn5, rrn4.5, rrn16, rrn23* |
| Transfer RNA | *trnA-UGC*^a^*, trnC-GCA, trnD-GUC, trnE-UUC, trnF-GAA, trnG-GCC, trnG-UCC*^a^*, trnH-GUG, trnI-CAU, trnI-GAU*^a^*, trnK-UUU*^a^*, trnL-CAA, trnL-UAA*^a^*, trnL-UAG, trnfM-CAU, trnM-CAU, trnN-GUU, trnP-UGG,trnQ-UUG, trnR-ACG, trnR-UCU, trnS-GCU, trnS-GGA, trnS-UGA, trnT-GGU, trnT-UGU, trnV-GAC, trnV-UAC*^a^*, trnW-CCA, trnY-GUA* |
| Other genes |  |
| RNA processing | *matK* |
| Carbon metabolism | *cemA* |
| Fatty acid synthesis | *accD* |
| Proteolysis | *clpP*^b^ |
| Genes of unknown function |  |
| Conserved reading frames | *ycf1, ycf2* |
| Note: ^a^ indicates the genes containing a single intron; ^b^ indicates the genes containing two introns. | |
